# The Relation of Pregnancy to Fibromyomata of the Uterus

**Published:** 1908-06-13

**Authors:** 


### OBSTETRICS.

### THE RELATION OF PREGNANCY TO FIBROMYOMATA OF THE UTERUS.

It has long been a question whether uterine fibroids prevent conception, or whether the absence of pregnancy predisposes to the growth of fibroids. Incidentally, the frequency of occurrence of fibroids in married or single women has some bearing on the point, and all are not even agreed upon this. Some observers think that single women are more subject to fibroids than married ones, whereas the reverse is held by others. The former believe that the absence of pregnancy predisposes to the growth of fibroids, the latter that the pregnant state encourages their growth, or, at least, that sexual relations have a bearing upon it.

There is a good deal of evidence from the purely clinical side that married women are more liable than others to fibromyomata. Thus, Schroeder noted 614 married women in 792 cases, and Winckel puts the proportion of married to single as two to one. On the other hand, of the married women who are the subjects of fibroids, a very large proportion are sterile, or, at least, have an unusually small number of children. Naturally, this must depend somewhat upon the time at which the fibroid first makes its appearance. In many cases the patients have had children before the growth appears, and then become sterile apparently in consequence thereof. It cannot in the least be asserted that fibroids always lead to sterility, because every practitioner knows of cases where the two conditions have been coincident. The fact seems to be that the situation and nature of the tumour have a very important bearing on its effect upon conception. For instance, cervical fibroids do not prevent conception, nor, as a rule, do subperitoneal growths. On the other hand, intestinal and especially submucous fibroids have a marked effect in preventing conception, just as they always affect the endometrium and cause menorrhagia.

There are several reasons, some of which are purely hypothetical, why fibroids should prevent conception. First among them is the enlargement of the uterine cavity found in the interstitial, submucous or polypoid sorts, which may prevent the arrest of a fertilised ovum because the walls are not in contact. (2) The presence of endometritis, with a thickened mucous membrane, may prevent conception by reason of constant discharges, which may have a deleterious effect upon spermatozoa, or may cause relative sterility, because endometritis so readily causes abortion. (3) The mucous membrane over the tumour and uterine wall may be atrophied from pressure, and so a fertilised ovum may fail to embed itself. (4) The occasional co-existence of tubal inflammation with fibroids may lead to occlusion of the fimbriated extremity, and so prevent the entrance of an ovum, or stretching and distortion of the tube over the tumour may obstruct its lumen.

From these considerations it is at least probable that fibroids have a distinct effect in preventing conception. On the other hand, the theory that sterility predisposes to the growth of fibroids rests upon grounds which are purely hypothetical. The suggestion is that new growths are apt to occur in disused organs, or organs that are not carrying out their complete functions. This theory is based on the belief that single women suffer more from fibroids, but the weight of evidence is against this. We are, therefore, bound to conclude on the whole that fibromyomata of the uterus do have a distinct effect in rendering conception difficult, but that sterility in no way disposes to the formation of these tumours.

When pregnancy has occurred in a uterus which also contains one or more fibroids, the due development of the embryo as a rule is not interfered with. On occasions, however, the peculiar situation of a fibroid interferes with the normal growth and dilatation of the uterus so that abortion occurs. Repeated abortion has been known to occur in some cases, and this is probably in some way connected with the prevention of due dilatation of the uterine cavity. The tumour itself, on the other hand, always undergoes hypertrophy during pregnancy. The increase in size is often quite notable, and is due to the same cause as the hypertrophy of the uterine wall. Such increase of a fibroid depends somewhat on the situation of the tumour, and the ultimate fate of the fibroid may be different from what would naturally be expected. In some cases the tumour undergoes involution after labour, and becomes the same size as it was before pregnancy. In others, however, the tumour undergoes necrobiosis, a form of aseptic tissue death. This pathological change is considered to be the result of some change in the blood-supply of the tumour, probably brought about by the ever-recurring uterine contractions during pregnancy, and the powerful contractions of labour. The amount of necrobiosis is variable; sometimes the whole tumour dies *en masse*; sometimes only portions are affected. In any case, the effect upon the patient is important, for this change of the tumour causes pain and rises of temperature. For these reasons it is often a sufficient cause for radical treatment to be adopted. When removed the necrobiotic fibroid is easily recognised because the dead portions have the red colour of raw beef, and are said to emit a fishy odour. It has been asserted, but on quite insufficient grounds, that fibroids sometimes disappear after pregnancy. However, in the light of modern teaching with regard to necrobiosis, it certainly appears that some fibroids may possibly be absorbed. If a small tumour underwent necrobiosis there is no *a priori* reason why it should not be absorbed as a result of a phagocytic process.

